# Activities of aztreonam in combination with several novel β-lactam-β-lactamase inhibitor combinations against carbapenem-resistant *Klebsiella pneumoniae* strains coproducing KPC and NDM

**DOI:** 10.3389/fmicb.2024.1210313

**Published:** 2024-03-05

**Authors:** Xinhui Li, Jisheng Zhang, Jianmin Wang, Wenzhang Long, Xushan Liang, Yang Yang, Xue Gong, Jie Li, Longjin Liu, Xiaoli Zhang

**Affiliations:** Department of Microbiology, Yongchuan Hospital of Chongqing Medical University, Chongqing, China

**Keywords:** carbapenem-resistant *Klebsiella pneumoniae*, β-lactamase inhibitor, bactericidal effect, time-kill assay, aztreonam

## Abstract

Isolates coproducing serine/metallo-carbapenems are a serious emerging public health threat, given their rapid dissemination and the limited number of treatment options. The purposes of this study were to evaluate the *in vitro* antibacterial activity of novel β-lactam-β-lactamase inhibitor combinations (BLBLIs) against carbapenem-resistant *Klebsiella pneumoniae* (CRKP) coproducing metallo-β-lactamase and serine-β-lactamase, and to explore their effects in combination with aztreonam, meropenem, or polymyxin in order to identify the best therapeutic options. Four CRKP isolates coproducing *K. pneumoniae* carbapenemase (KPC) and New Delhi metallo-β-lactamase (NDM) were selected, and a microdilution broth method was used to determine their susceptibility to antibiotics. Time-kill assay was used to detect the bactericidal effects of the combinations of antibiotics. The minimum inhibitory concentration (MIC) values for imipenem and meropenem in three isolates did not decrease after the addition of relebactam or varbobactam, but the addition of avibactam to aztreonam reduced the MIC by more than 64-fold. Time-kill assay demonstrated that imipenem-cilastatin/relebactam (ICR) alone exerted a bacteriostatic effect against three isolates (average reduction: 1.88 log_10_ CFU/mL) and ICR combined with aztreonam exerted an additive effect. Aztreonam combined with meropenem/varbobactam (MEV) or ceftazidime/avibactam (CZA) showed synergistic effects, while the effect of aztreonam combined with CZA was inferior to that of MEV. Compared with the same concentration of aztreonam plus CZA combination, aztreonam/avibactam had a better bactericidal effect (24 h bacterial count reduction >3 log_10_CFU/mL). These data indicate that the combination of ATM with several new BLBLIs exerts powerful bactericidal activity, which suggests that these double β-lactam combinations might provide potential alternative treatments for infections caused by pathogens coproducing-serine/metallo-carbapenems.

## Introduction

The emergence and widespread dissemination of acquired β-lactamase genes have made carbapenem-resistant Enterobacterales (CRE) a formidable challenge in global public health and clinical management (Gandra and Burnham, 2020). Meanwhile, carbapenem-resistant *Klebsiella pneumoniae* (CRKP) causes severe infections in debilitated and immunocompromised patients, leading to extended hospital stays and increased mortality, and has been classified as an “urgent threat” by the US Centers for Disease Control and Prevention (CDC) (Tzouvelekis et al., 2012). Resistance of CRKP to β-lactam antibiotics is usually associated with the production of β-lactamases, including extended-spectrum β-lactamases (ESBLs) and carbapenemases belonging to various molecular classes (Nordmann and Poirel, 2019). To counteract the hydrolytic activity of these enzymes and to restore the antimicrobial activity of some β-lactam antibiotics, combinations of β-lactam with β-lactamase inhibitor (BLI) have been developed; this represented a breakthrough for clinical treatment, and some of these combinations have been approved by the Food and Drug Administration (FDA). The main novel groups are diazobicyclooctanes (DBOs) (avibactam and relebactam) and boronic acid derivatives (vaborbactam) (Yahav et al., 2020). Avibactam has been used in combination with ceftazidime, which provides extensive activity against Enterobacterales and *Pseudomonas aeruginosa* expressing one or multiple β-lactamases (Zhanel et al., 2013; Castanheira et al., 2015). Relebactam, which is closely related to avibactam at the structural level, was developed to enhance the activity of imipenem after displaying compatibility and effectiveness both *in vitro* and in mouse infection models of CRE (Blizzard et al., 2014; Papp-Wallace et al., 2018). A multicenter comparative study found that imipenem-cilastatin/relebactam (ICR) was more effective than imipenem plus polymyxin therapy and was an efficacious and well-tolerated treatment option for carbapenem-resistant infections (Motsch et al., 2020). Vaborbactam was approved for use in combination with meropenem [meropenem/vaborbactam (MEV)] by the FDA in August 2017, showing excellent *in vitro* activity against KPC-producing Enterobacterales (Hackel et al., 2018). Although these combinations are very active against class A β-lactamase-producing Enterobacterales, none of them display activity against metallo-β-lactamase (MBL)-producing isolates.

Aztreonam remains a clinically available agent for metallo-β-lactamase-producing strains due to its ability to evade MBL-mediated hydrolysis, but it can be hydrolyzed by most clinically relevant serine beta-lactamases, such as ESBLs, AmpC, and KPC (Brogden and Heel, 1986). The most troubling isolates are probably those that produce both serinase and MBLs, since most β-lactam antibiotics and BLI inhibitors are ineffective against them. In recent years, more and more studies have sought to use combination therapy treatment against MBL-producing strains, and some of these have indicated that a combination of aztreonam plus CZA displays *in vitro* synergy against MBL-producing Enterobacterales (Marshall et al., 2017; Jayol et al., 2018). It has also been found that the combination of aztreonam plus avibactam is highly active against MBL-producing strains, with the MIC_90_ being 0.5 mg/L in a wide range of CREs (Karlowsky et al., 2017). This raises the question of whether a combination of aztreonam with other β-lactam-β-lactamase inhibitor combinations (BLBLIs) is a potential treatment option against strains coproducing serinase and MBL. In this study, to explore the best potential treatment combination against strains coproducing serine-β-lactamase and MBL, time-kill assay was used to assess and compare the antibacterial effects of several combinations of aztreonam with antibiotics (including CZA, MEV, ICR, AVI, polymyxin, and meropenem).

## Materials and methods

### Bacterial strains and resistance characteristics

A total of 127 CRKP isolates were collected from patients admitted to Yongchuan Hospital of Chongqing Medical University from 2019 to 2020. Identification at the species level was performed by matrix-assisted laser desorption/ionization–time-of-flight (MALDI-TOF) mass spectrometry (Bruker Daltonik, Bremen, Germany) and by analysis using a VITEK-2 automated microbiology analyzer (bioMérieux, France). A modified carbapenem inactivation method (mCIM) and an EDTA-modified carbapenem inactivation method (eCIM) were used to conduct preliminary screening of the production of carbapemase, as described in the Supplementary materials. The beta-lactamase genes *bla*_CTX-M_, *bla*_SHV_, *bla*_TEM_, *bla*_OXA_, *bla*_KPC_, and *bla*_NDM_ were routinely amplified via PCR, and the positive results were sequenced via Sanger sequencing. All primers were obtained from previous studies (Gong et al., 2018; Huang et al., 2021). The patient electronic medical records corresponding to all strains were collected from the hospital information management system and the laboratory information management system of our hospital. Four *K. pneumoniae* clinical isolates that were coproducers of KPC and NDM (CRKP238, CRKP241, CRKP279, and CRKP319) were obtained and utilized for all experiments.

### Multilocus sequence typing (MLST)

Multilocus sequence typing (MLST) was performed using seven housekeeping genes of *K. pneumoniae* that were amplified using primers from online databases,^1^ and sequence types (STs) were determined using online database tools. The novel allele profiles were sent to klebsiellaMLST@pasteur.fr for confirmation.

### Antimicrobial susceptibility testing

The antibiotics tested in this study were tigecycline, polymyxin, meropenem, imipenem, amikacin, levofloxacin, aztreonam, CZA, MEV, ICR, and aztreonam/avibactam. All antibiotic powders were weighed in an electronic balance and prepared in stock antibiotic solutions at all storage concentrations in sterile water (DMSO for aztreonam) and stored at −80°C. The specific dissolution method is shown in Supplementary Table S1. Subsequently, the aliquots were thawed and diluted to the desired concentrations with cation-adjusted Mueller Hinton broth (CAMHB). Antimicrobial susceptibility was evaluated using reference broth microdilution methods, conducted according to Clinical and Laboratory Standards Institute (CLSI) procedures (document M07). Quality control strains included *Escherichia coli* ATCC 25922, *K. pneumoniae* ATCC 700603, and ATCC BAA-1705, and the minimum inhibitory concentrations (MICs) were interpreted according to CLSI recommendations.

### Time-kill experiment

Time-kill studies were performed to analyze the bactericidal activity of the selected antibiotics alone and in combination with BLBLIs at clinically achievable free-drug concentrations. All experiments were performed in duplicate. An overnight culture of isolate was diluted with LB broth and further incubated at 37°C (120 rpm) for 12 h to reach early log-phase growth. The initial bacterial inoculum was adjusted to 10^6^CFU/mL in fresh CAMHB broth. Antibiotic concentrations used during time-kill experiments represent mean steady-state concentrations of non-protein-bound drug in humans, as calculated from data in the literature (based on the area under the antibiotic concentration–time curve in serum or plasma over 24 h divided by 24 h [AUC_0–24_/24 h]). The following antibiotic concentrations were used: aztreonam, 17 mg/L (Tangden et al., 2014); meropenem, 10 mg/L (Benitez-Cano et al., 2020); polymyxin, 2 mg/L (Tsuji et al., 2019); CZA, 33.5/6 mg/L (Das et al., 2020); aztreonam/avibactam, 17/6 mg/L; MEV, 23.2/25.5 mg/L (Wenzler et al., 2015); and ICR, 11.4/7.5 mg/L (Rizk et al., 2018). Viable colony counts were performed by obtaining samples after 0, 2, 4, 8, 12, and 24 h of antibiotic exposure. Synergy was defined as induction of a reduction by ≥2 log_10_ CFU/mL by the combination compared with the most active agent alone. Bactericidal activity was defined as a ≥ 3 log_10_ CFU/mL reduction in viable bacterial count at 24 h compared with the initial inoculum. The detection limit of the time-kill assay was 2.17 log_10_ CFU/mL.

## Results

### Bacterial strains and patient characteristics

**Figure 1:**
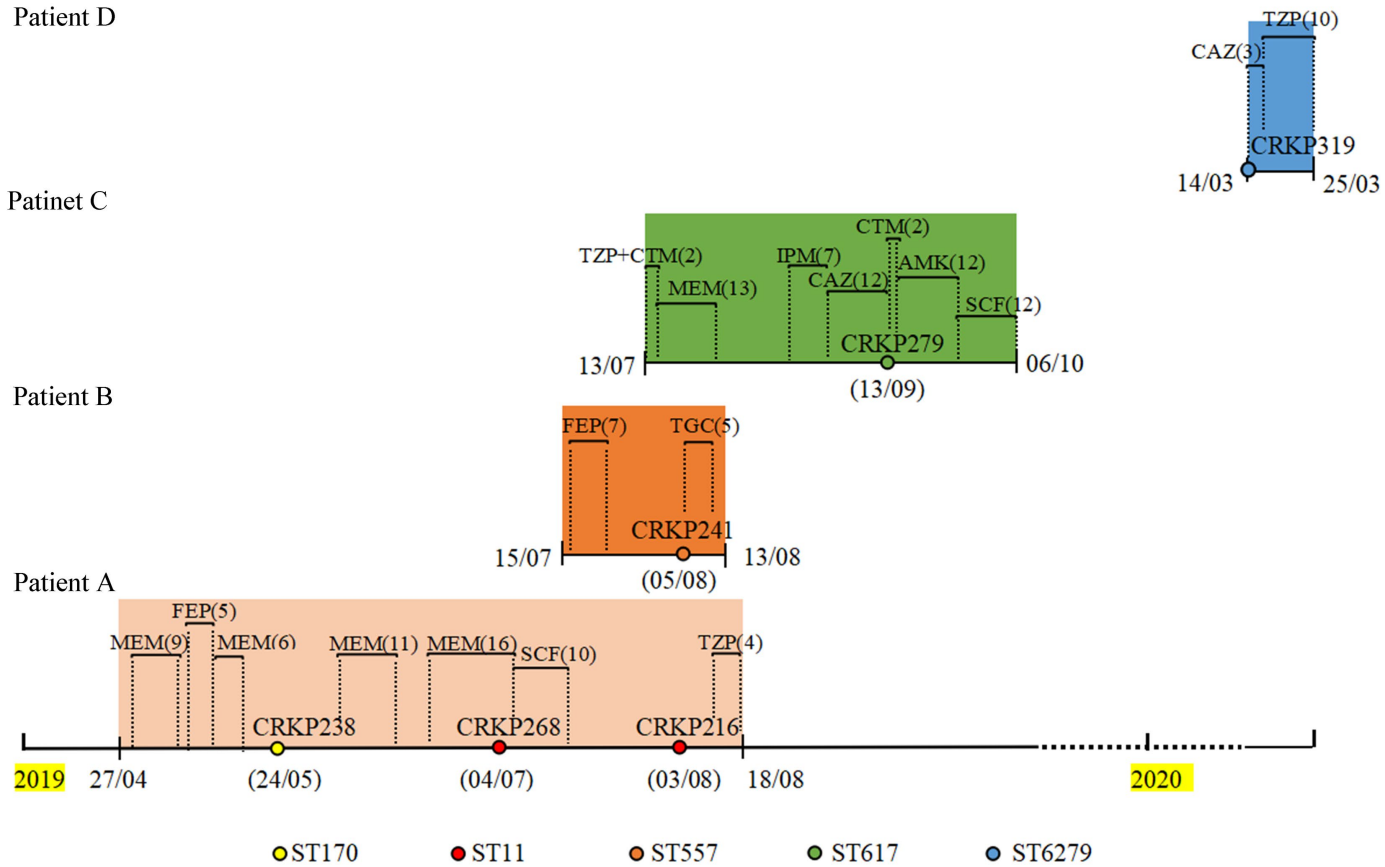
Timeline of CRKP infection and treatments during the patients’ hospitalizations. CRKP238, CRKP268, CRKP216, CRKP241, CRKP279, and CRKP319 are carbapenem-resistant *Klebsiella pneumoniae* strains. MEM, meropenem; FEP, cefepime; SCF, cefoperazone/sulbactam; TZP, piperacillin/tazobactam; TGC, Tigecycline; CTM, cefotiam; IPM, imipenem; CAZ, ceftazidime; AMK, amikacin.

The CZA resistance rate of 127 CRKP isolates collected from 2019 to 2020 was 12.5% (16/127). The resistance and carbapenemase production status of these isolates are shown in Supplementary Table S2; among these, four CZA-resistant isolates contained NDM enzyme and KPC enzyme, nine isolates only produced NDM enzyme, one isolate produced IMP enzyme, and two isolates only produced KPC-2 enzyme. The treatment history of patients with isolates coproducing NDM and KPC was further analyzed. As shown in Figure 1, three strains (CRKP238, CRKP268, and CRKP216) were isolated from patient A, who was admitted to the respiratory critical care unit due to pneumonia. After a period of treatment with meropenem and cefepime, an ST170 isolate (CRKP238) carrying *bla*_KPC-2_ and *bla*_NDM-5_ was isolated from sputum. After the patient was transferred to the respiratory intermediate care unit (RICU) and had continued to be treated with meropenem and cefoperazone/sulbactam for a period of time, two ST11 isolates (CRKP268 and CRKP216) that only produced KPC-2 enzyme were isolated in the urine and secretions, respectively. Patient B was admitted to the respiratory critical care department due to chronic obstructive pulmonary disease (COPD). After 7 days of treatment with cefepime, CRKP241 was isolated from the patient; he was then discharged after 5 days of treatment with tigecycline. Patient C, a preterm infant, was treated with meropenem, imipenem, and ceftazidime, after which a multidrug-resistant isolate (CRKP279) was isolated from sputum. After treatment with amikacin cefoperazone/sulbactam, patient C was cured and discharged. Finally, CRKP319 belongs to ST6279, which is a novel ST identified in our study, exhibiting a multidrug resistance phenotype; it was isolated from patient D, with neonatal pneumonia, who was discharged after treatment with ceftazidime and cefoperazone/sulbactam.

### Antimicrobial susceptibility testing

As shown in Table 1, the four isolates selected in this study carried *bla*_TEM_, *bla*_KPC-2_, and *bla*_NDM-1_ or *bla*_NDM-5_, respectively. In addition, CRKP238 also produced *bla*_CTX-M-65_. Four strains were resistant to meropenem and imipenem; addition of neither 4 mg/L relebactam nor 8 mg/L vaborbactam resulted in a decrease in the MIC values, except in the case of CRKP238. For CRKP238, adding relebactam to imipenem reduced the MIC value by 256-fold, and adding vaborbactam to meropenem reduced the MIC value by more than 128-fold. All strains were highly resistant to CZA (MIC >256 mg/L) and aztreonam (MIC≥32 mg/L). While the addition of 4 mg/L avibactam to aztreonam significantly decreased the MIC by more than 64-fold, MIC values of three of the strains against polymyxin were 2 mg/L, the exception being CRKP241, which was resistant to polymyxin.

**Table 1:** The minimum inhibitory concentrations (MICs) of antibiotics and antimicrobial resistance genes (ARGs) of four carbapenem-resistant *Klebsiella pneumoniae* isolates.

Antibiotics	CRKP238	CRKP241	CRKP279	CRKP319
MIC (mg/L)				
Imipenem	64	8	16	16
Meropenem	>128	16	64	32
Amikacin	2	2	2	>128
Levofloxacin	64	4	<0.125	16
Tigecycline	2	32	2	4
Polymyxin	2	4	2	2
Aztreonam	>128	>128	32	>128
ATM/AVI	0.5/4	0.5/4	0.5/4	0.25/4
CZA	>256/4	>256/4	>256/4	>256/4
ICR	0.25/4	8/4	32/4	16/4
MEV	1/8	16/8	>32/8	16/8
ARGs	*bla*_KPC-2_ *bla*_NDM-5_ *bla*_TEM_ *bla*_CTX-M-65_	*bla*_KPC-2_ *bla*_NDM-1_ *bla*_TEM_ *qnrS*	*bla*_KPC-2_ *bla*_NDM-5_ *bla*_TEM_	*bla*_KPC-2_ *bla*_NDM-1_ *bla*_TEM_ *bla*_SHV_ *qnrS*

ATM/AVI, aztreonam/avibactam; CZA, ceftazidime/avibactam; ICR, imipenem-cilastatin/relebactam; MEV, meropenem/vaborbactam.

### Time-kill assay results

**Figure 2:**
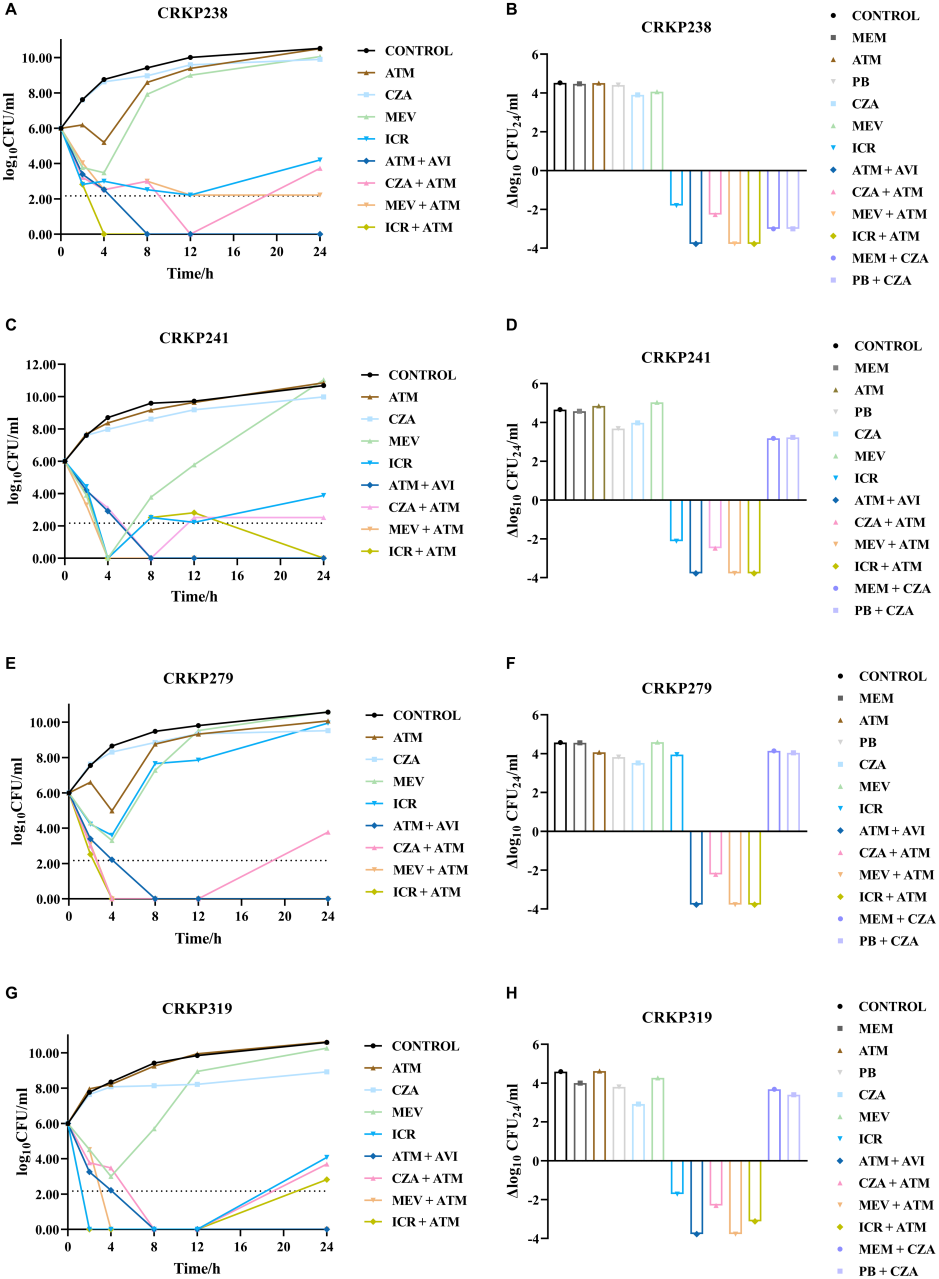
Bacterial load (log10 CFU/mL) over the course of 24 h in the four carbapenem-resistant *Klebsiella pneumoniae* isolates coproducing KPC and NDM for each antibiotic combination regimen were shown in **A–H**. LOD (lower limit of detection) = 2.17 log10 CFU/mL. ATM, aztreonam; CZA, ceftazidime/avibactam; MEV, meropenem/vaborbactam; ICR, imipenem-cilastatin/relebactam; AVI, avibactam; MEM, meropenem; PB, polymyxin.

The growth and kill trends for four isolates cultured with seven antibiotics at average steady-state serum concentrations are shown in Figure 2; Supplementary Figure S1. Bacterial growth without antibiotics reached 10 to 11 log_10_ CFU/mL at 24 h for all isolates. Single antibiotics (aztreonam, meropenem, and polymyxin) were not bactericidal against any of the isolates at 24 h. CZA and MEV monotherapy were also not bactericidal against any of the isolates at 24 h. The CZA combination therapies with different antibiotics showed different effects as compared with monotherapy. Neither CZA plus meropenem nor CZA plus polymyxin was bactericidal against CRKP241, CRKP279, or CRKP319. For CRKP238, CZA combined with meropenem or polymyxin achieved more than 3 log_10_ CFU/mL reduction compared with the initial inoculum (Figure 2; Supplementary Figure S1). Although aztreonam alone was ineffective against all four strains, it had a synergistic effect when combined with CZA. The combination of aztreonam plus CZA produced bactericidal activity at 12 h, which achieved more than 3.78 log_10_ CFU/mL bacteria reduction, except in the case of strain CRKP241. However, in all isolates, regrowth was observed at 24 h, with an average reduction of 2.30 log_10_ CFU/mL compared with the initial inoculum.

Similar to CZA monotherapy, the use of MEV alone produced no bactericidal activity against any strains at 24 h. However, the combination of MEV plus aztreonam had a synergistic effect, resulting in more than 3.78 log10 CFU/mL reduction at 24 h. Unlike CZA and MEV monotherapy, ICR alone decreased bacteria relative to the inoculum to varying degrees at 24 h (for CRKP238, 1.8 log_10_CFU/mL reduction; for CRKP241, approximately 2.12 log_10_ CFU/mL reduction; for CRKP319, approximately 1.72 log_10_ CFU/mL reduction). However, ICR plus aztreonam produced bactericidal activity against all strains, but this effect was only an additive one. In addition, the combination of aztreonam and avibactam tested in this study was found to exhibit bactericidal activity and reduced the amount of bacteria by more than 3.78 log_10_ CFU/mL.

## Discussion

β-lactam antibiotics are the most widely used and abundant antibiotics in clinical practice. Unfortunately, the abuse of carbapenems has undoubtedly contributed to the emergence and widespread distribution of acquired β-lactamase genes, which have led to an increasing resistance rate. In the case of serine/metallo-enzyme-coproducing pathogens, often referred to as “superbugs,” which are capable of hydrolyzing nearly all available β-lactam antibiotics, BLBLI preparations may not be effective in terms of their bactericidal effect, and there is an ongoing quest for new treatment.

It was expected that no bactericidal effect would be observed for CZA and MEV monotherapy, although the MIC values against MEV were lower than the concentrations used in the time-kill assay, except in the case of CRKP279. Published data show that ICR has excellent *in vitro* activity against isolates carrying class A and C β-lactamases (Hirsch et al., 2012) but is not effective in restoring susceptibility to isolates expressing OXA-48 or MBL carbapenemases (Lapuebla et al., 2015). However, recent data from a small sample showed that 42.9% of MBL-producing *K. pneumoniae* isolates (6/14) were susceptible to ICR (Yang et al., 2022). Contrary to expectations, ICR showed bacteriostatic activity against three strains in this study, although the addition of relebactam to imipenem did not reduce the MIC against three of the strains, with CRKP238 being the exception. In a study by Yu et al., ICR displayed bactericidal activity against four MBL-producing isolates at 24 h (Yu et al., 2021); the amount of bacteria in three isolates producing NDM or IMP enzyme decreased by >5 log_10_ CFU/mL. These observations highlight a need to further evaluate the effectiveness of ICR against MBL-producing isolates.

Aztreonam is a special β-lactam, given its resistance to MBL inhibition, while the presence of KPC can hydrolyze it, so aztreonam alone is not enough to fight against strains coproducing MBL and KPC enzyme. The synergistic effects of aztreonam plus CZA or MEV have been confirmed *in vitro* and *in vivo* (Marshall et al., 2017; Biagi et al., 2019), and a recent observational study indicated that the combination of CZA plus aztreonam offers a therapeutic advantage for patients with bloodstream infections due to MBL-producing Enterobacterales (Falcone et al., 2021). Our results demonstrated that the combinations of aztreonam plus CZA or MEV both had a synergistic effect, whereas MEV combinations had better bactericidal activity at 24 h compared with CZA combinations. In addition, bacterial regrowth was observed in three strains at 24 h, which suggests that clinical killing of bacteria may be possible by increasing the frequency of drug administration. We also found that the bactericidal effect achieved by the combination of aztreonam plus ICR was similar to that of aztreonam plus MEV, which can reduce the amount of bacteria by more than 3.78 log_10_ CFU/mL.

Avibactam is also currently in development in combination with aztreonam; although avibactam does not inhibit MBLs, this combination restores the activity of aztreonam against MBL-producing pathogens via inhibition of co-expressed serinase. The aztreonam/avibactam combination has demonstrated potent *in vitro* activity against MBL-producing Enterobacterales in several surveillance studies. In the global INFORM surveillance study, the MIC_50/90_ against OXA-48 plus MBL (n = 23) was 0.25/0.5 mg/L, respectively (Kazmierczak et al., 2018). Our *in vitro* susceptibility test (MIC values ≤0.5/4 mg/L) and time-kill assay also showed that aztreonam/avibactam was potent against CRKPs coproducing NDM and KPC.

While the theory behind combining aztreonam with avibactam, CZA, MEV, or ICR is the same, notable differences between these combinations are present in the form of the penicillin-binding protein (PBP) targets of the β-lactams and the β-lactamase affinity of the inhibitors, which may lead to different effects. Aztreonam has high affinity for PBP3, while ceftazidime mainly binds to PBP1a/b and PBP3, disrupting peptidoglycan synthesis in *K. pneumoniae* (Sutaria et al., 2018). In this study, the time-kill assay results demonstrated that aztreonam/avibactam was more effective than aztreonam plus CZA, reducing the amount of bacteria to a greater extent. We speculate that ceftazidime and aztreonam competitively bind to PBP3, while ceftazidime can be hydrolyzed by NDM to counteract its bactericidal effect, resulting in the inferior combined effect of aztreonam plus CZA as compared to aztreonam/avibactam. It has been found that the saturation of one or more PBPs also results in different bacterial dissolution rates, and the saturation of multiple PBP sites leads to higher bacterial dissolution rates (Satta et al., 1995). Meropenem and imipenem bind to PBP2 and PBP4 (Sutaria et al., 2018), and when these are combined with aztreonam, the combination could lead to the saturation of multiple sites, which may explain the better combined effect of MEV or ICR plus aztreonam in this experiment. In view of the different bactericidal effects of these double β-lactam combinations, promising dual-drug and triple-drug combination administration strategies can be rationally designed and optimized in the future based on existing PBP binding sites and β-lactamase mechanisms against multidrug-resistant *K. pneumoniae*, which may be superior to unoptimized, empirical double β-lactam combinations.

Different effects have been observed for the combination of polymyxin plus CZA. Some experiments have found that this combination does not improve the survival rate of *Galleria mellonella* (Borjan et al., 2020), while others have found that the combination of colistin plus CZA has a synergistic bactericidal effect against MBL-producing strains (Montero et al., 2021). In addition, one study tested the efficacy of a double β-lactam strategy against carbapenemase-producing isolates, finding that CZA combined with meropenem or imipenem showed synergy against certain KPC-producing strains (Gaibani et al., 2017). However, our results only detected one strain that showed synergistic effect in time-kill assay. The effects of meropenem or polymyxin combined with CZA need further investigation.

## Conclusion

The combination of aztreonam plus avibactam, CZA, MEV, and ICR showed good antibacterial activity. These double β-lactam combinations offer potential solutions for isolates coproducing MBL and KPC. However, the collection in this study was monocentric, and the 24-h static nature of *in vitro* time-kill experiments is a limitation of this study. More isolates are needed to conduct *in vitro* and *in vivo* studies to further determine the appropriate selection of and optimal dosing regimen for novel agents.

## Data availability statement

The datasets presented in this study can be found in online repositories. The names of the repository/repositories and accession number(s) can be found in the article/Supplementary material.

## Ethics statement

The studies involving humans were approved by the Ethics Committee of Yongchuan Hospital of Chongqing Medical University. The studies were conducted in accordance with the local legislation and institutional requirements. The human samples used in this study were primarily isolated as part of a previous study, for which ethical approval was obtained. Written informed consent for participation was not required from the participants or the participants’ legal guardians/next of kin in accordance with the national legislation and institutional requirements.

## Author contributions

XZ, XiL, JW, and JZ designed the study. XiL and LL performed the experiments. YY, XG, and JL analyzed and interpreted the data, collected the isolates, and the clinical data. XiL and XZ drafted the manuscript. XZ reviewed the manuscript and provided recommendations. All authors contributed to the article and approved the submitted version.

## Footnotes

^1^ https://bigsdb.pasteur.fr/klebsiella/primers-used/
