# Supplementary material for: Activities of aztreonam in combination with several novel β-lactam-β-lactamase inhibitor combinations against carbapenem-resistant Klebsiella pneumoniae strains coproducing KPC and NDM
Source: Front Microbiol. 2024 Mar 5;15:1210313. doi: 10.3389/fmicb.2024.1210313 (PMC10949892; doi:10.3389/fmicb.2024.1210313)
Supplement: Supplementary file 1 [file Table_1.DOCX]

**Supplementary materials**

**Methods**

mCIM and EDTA-modified mCIM detailed methods

mCIM was performed according to the document M100-S27 by the CLSI. Briefly, 2 ml aliquots of trypticase soy broth (TSB) were directly inoculated with a 1μl loopful of CRKP colonies. The suspension was vortexed and a 10 μg meropenem disk (Oxoid) was placed in the inoculated tube. About 0.5 McFarland bacterial suspension of meropenem-susceptible E.coli ATCC 25922 were prepared to placed on a Mueller–Hinton agar (MHA). Tubes were incubated for 4 h (± 15 min), then meropenem disk was removed from the inoculated tube and attached to the MHA plate with ATCC 25922 plated on it. After incubation at 35℃ for 18-24h, the diameter of antibacterial zone was measured.

For eCIM testing, each isolate was inoculated in a 2 ml aliquot of TSB-EDTA (EDTA concentration, 5 mM). Isolates were incubated and plated as described for mCIM testing. Plates were incubated for 18 to 24 h, and the interpretation for both assays was according to CLSI-2020. BAA-1705 and BAA-1706 were used as control strains.

mCIM result interpretation

Carbapenemase positive: inhibition zone diameter between 6-15mm or inhibition zone diameter between 16-18mm but there were colonies scattered within it. Carbapenemase negative: inhibition zone diameter ≥19mm. Carbapenemase Intermediate: the diameter of the inhibition zone was 16-18mm, or the diameter was ≥19mm but there were scattered colonies in the inhibition zone; The presence or absence of carbapenase could not be determined.

eCIM result interpretation

Carbapenemase positive:

Compared with the results of mCIM, the antibacterial zone diameter was more than 5mm. Metallo-enzyme negative: Compared with mCIM results, the diameter of the inhibition zone is less than 4mm.

Table S1. Solvents, diluents, and storage concentrations for preparing stock solutions of antimicrobial agentsa.

| Antimicrobial Agent | Solvent | Diluent | Storage concentrations |
| --- | --- | --- | --- |
| Tigecycline | water | water | 50mg/mL |
| Polymyxin | water | water | 10μg/mL |
| Meropenem | water | water | 5mg/mL |
| Amikacin | water | water | 100mg/mL |
| Aztreonam | DMSO | watar | 50mg/mL |
| Ceftazidime | water | water | 50mg/mL |
| Vaborbactam | water | water | 2mg/mL |
| Relebactam | water | water | 5mg/mL |
| Avibactam | water | water | 10mg/mL |

The solvents of antibacterials mainly refer to the agents instructions and CLSI documents. The actual storage concentration of the antibacterials is setted according to the instructions and the specific dissolution condition.
