# Supplementary material for: Activities of aztreonam in combination with several novel β-lactam-β-lactamase inhibitor combinations against carbapenem-resistant Klebsiella pneumoniae strains coproducing KPC and NDM
Source: Front Microbiol. 2024 Mar 5;15:1210313. doi: 10.3389/fmicb.2024.1210313 (PMC10949892; doi:10.3389/fmicb.2024.1210313)
Supplement: Supplementary file 2 [file Table_2.DOCX]

| Number of isolates | Resistance gene | ST |
| --- | --- | --- |
| CRKP207 | *bla*_NDM-5_ | 258 |
| CRKP 213 | *bla*_NDM-1_ | 1887 |
| CRKP 221 | *bla*_NDM-5_ | 29 |
| CRKP 225 | *bla*_KPC-2_ | 11 |
| CRKP 238 | *bla*_KPC-2_、*bla*_NDM-5_ | 170 |
| CRKP 239 | *bla*_NDM-5_ | 617 |
| CRKP 241 | *bla*_KPC-2_ 、*bla*_NDM-1_ | 557 |
| CRKP 263 | *bla*_KPC-2_ | 11 |
| CRKP 276 | *bla*_NDM-5_ | 617 |
| CRKP 279 | *bla*_KPC-2_ 、*bla*_NDM-5_ | 517 |
| CRKP 294 | *bla*_IMP-4_ | 37 |
| CRKP 299 | *bla*_NDM-5_ | 617 |
| CRKP 319 | *bla*_KPC-2_ *、bla*_NDM-1_ | 6279 |
| CRKP 320 | *bla*_NDM-1_ | 1887 |
| CRKP 321 | *bla*_NDM-1_ | 1887 |
| CRKP 323 | *bla*_NDM-1_ | 1887 |

Table S2. Carbapenemases-producing characteristics of CZA-resistance strains.
