# Supplementary material for: Activities of aztreonam in combination with several novel β-lactam-β-lactamase inhibitor combinations against carbapenem-resistant Klebsiella pneumoniae strains coproducing KPC and NDM
Source: Front Microbiol. 2024 Mar 5;15:1210313. doi: 10.3389/fmicb.2024.1210313 (PMC10949892; doi:10.3389/fmicb.2024.1210313)
Supplement: Supplementary file 3 [file Data_Sheet_1.docx]

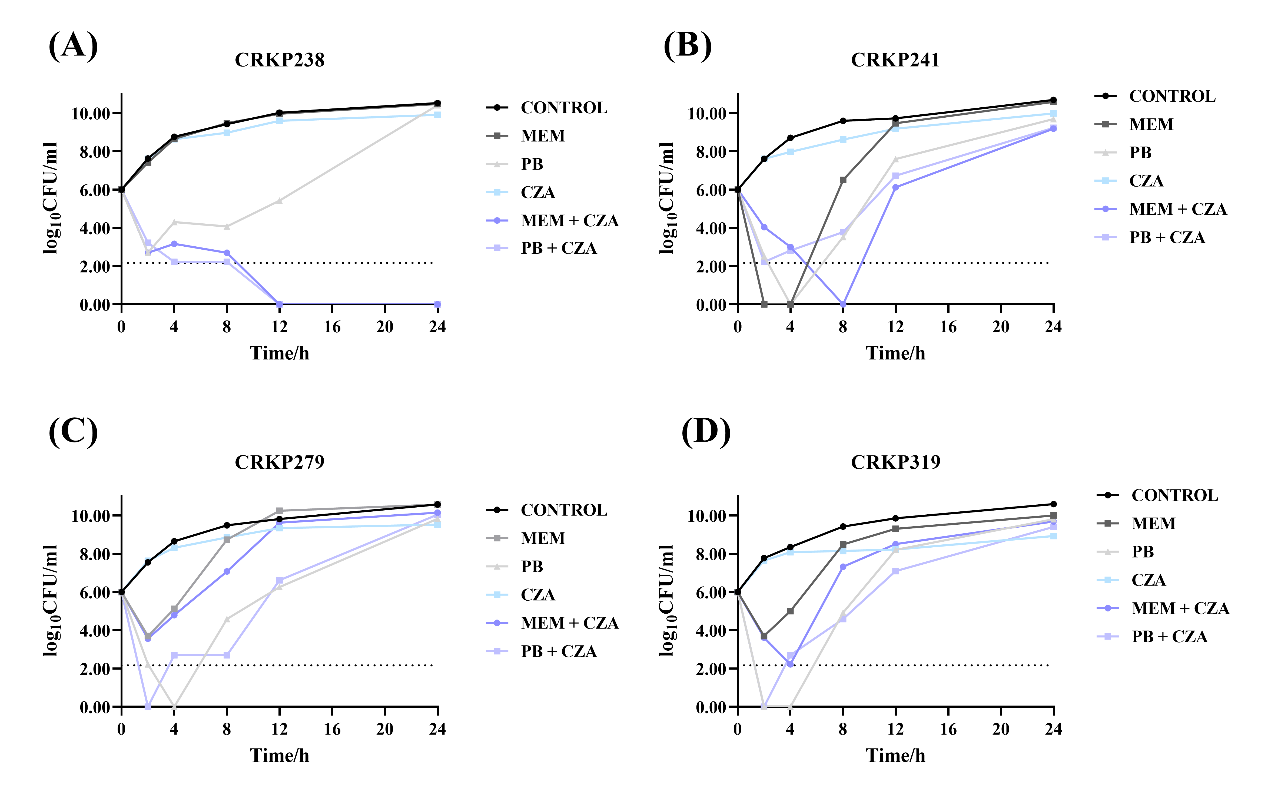


Figure S1. Bacterial load (log_10_ CFU/ml) over 24 h in the four KPC and NDM coproducing carbapenem-resistant *Klebsiella pneumoniae* isolates for PB and MEM monotherapy and combinations. LOD (lower limit of detection) = 2.17 log_10_ CFU/ml.
